# Pharmacists’ perspectives on implementing pharmacist-managed anticoagulant clinics in Makkah Region Ministry of Health Hospitals: A qualitative study

**DOI:** 10.1371/journal.pone.0342079

**Published:** 2026-02-02

**Authors:** Wassan L. Zarei, Sarah M. Khayyat

**Affiliations:** 1 Pharmaceutical Care Administration, Clinical Pharmacy Department, King Fahad General Hospital, Ministry of Health, Jeddah, Saudi Arabia; 2 Pharmaceutical Practices Department, College of Pharmacy, Umm Al-Qura University, Makkah, Saudi Arabia; Yarmouk University, JORDAN

## Abstract

**Objectives:**

This study aimed to identify the pharmacists’ perspectives on the barriers and facilitators to implementing pharmacist-managed anticoagulation clinics (PMACs) in Ministry of Health (MOH) hospitals in the Makkah Region, Saudi Arabia.

**Methods:**

This study employed a qualitative, cross-sectional design. An interview guide was developed after a review of the literature and the adaptation of the Consolidated Framework for Implementation Research tool. Semi-structured interviews were conducted with licensed regular pharmacists, clinical pharmacists, and pharmaceutical care leaders who were working or had worked in MOH hospitals in the Makkah Region.

**Results:**

A total of 11 participants were interviewed. The barriers to implementing PMACs predominantly related to professional and interprofessional barriers (e.g., lack of leader support and staff expertise, physician resistance), organizational and operational barriers (e.g., lack of resources), and patient-related barriers (e.g., poor commitment to follow-up). The main facilitators to implementing PMAC services were associated with improving workforce capacity and training, leadership and interprofessional support, organizational and patient support mechanisms, and infrastructure and digital resources.

**Conclusion:**

The factors identified in this study can be used to develop potential strategic plans to improve existing PMAC services and extend their implementation across the country. Future research should aim to quantify the effectiveness of PMAC services on patients’ clinical/non-clinical outcomes. Positive findings could provide further evidence of the value of PMAC services, speed up their expansion, and ensure their sustainability.

## 1. Introduction

Managing patients with coagulation disorders is influenced by several factors, including the medication’s narrow therapeutic window, variability in dose-response relationships, communication gaps between patients and physicians regarding dosing, and issues with patient adherence [1]. To address these factors and overcome the complexities of anticoagulation management, many countries have developed care models, including patient-centered models that emphasize patient self-management and self-testing, as well as pharmacist-managed, pharmacist-assisted, and nurse-managed care models [1,2]. Running a pharmacist-managed anticoagulation clinic (PMAC) offers many benefits to patient health [1,3,4]. A systematic review comparing the quality of warfarin anticoagulation control between outpatient PMACs and routine medical care found that the PMAC had better anticoagulation control and lower bleeding, thromboembolic events, and healthcare utilization [4]. The PMAC also improved patient knowledge of warfarin use and international normalized ratio (INR) control, leading to high patient satisfaction with the service [5].

A previous study explored the motivations behind establishing the first PMAC in Saudi Arabia, detailing its implementation process and its impact on anticoagulation management, adverse events, and patient satisfaction [6]. The findings indicated that the clinic had a favorable influence on patient care, as evidenced by the increased number of patients with INRs within range and patient satisfaction scores. The PMAC reduced time pressures on physicians, allowing them to focus on other patient care issues. Further advantages of the PMAC included lower costs of care, fewer concurrent medication interactions, and fewer adverse medication-related events while treatment objectives were still met [6]. The clinic’s implementation resulted in the expansion of pharmacy services, with an increased possibility of improving patient care. For example, a prospective observational study conducted in an ambulatory care setting in Riyadh City found that patients followed in the PMAC had higher time in therapeutic range (TTR) than those observed in a physician-managed clinic [7]. A study in Abha City suggested that pharmacist interventions can improve patients’ health-related quality of life when taking warfarin. Thus, the involvement of competent pharmacy personnel should be prioritized for both routine and critical patient care [8]. Based on national and international evidence, the Clinical Pharmacy Department of the General Administration of Pharmaceutical Care at the Saudi Ministry of Health (MOH) established the Clinical Practical Guidance for Warfarin Monitoring [9]. However, despite improvements in anticoagulation management and the availability of different models of care worldwide, PMACs have not been widely adopted in MOH hospitals. For example, only one of the seven MOH hospitals in Jeddah has a PMAC, while the MOH hospitals in Makkah and Taif do not. This qualitative study, therefore, aimed to explore pharmacists’ perspectives on the barriers and facilitators to implementing PMACs within MOH hospitals in the Makkah Region, where PMACs remain largely absent despite national clinical guidance, representing a critical gap between policy intent and real-world practice.

## 2. Methods

### 2.1. Study design

This qualitative cross-sectional, mono-method study included interviews with semi-structured open-ended questions to describe the meaning and significance of the study participants’ experiences and perspectives. The study reporting was guided by the Consolidated Criteria for Reporting Qualitative Research [10]. An adapted grounded theory approach provided the theoretical framework for the study. Two other frameworks were also applied: the knowledge-to-action (KTA) framework and the Consolidated Framework for Implementation Research (CFIR) [11,12]. The KTA model was used to design the study. For instance, the Knowledge Creation component and the first stage of the Action Cycle were considered during the literature review to identify the problem and knowledge gap. Then, the study focused on two stages during the interviews: adapting knowledge to the local context and assessing barriers and facilitators to implementing PMACs [12]. The CFIR tool was adapted to prepare the interview guide, analyze the data, and identify potential barriers and facilitators for implementing PMACs [11].

### 2.2. Eligibility criteria

All participants were adults (aged ≥ 18 years), and no minors were involved in the study. Participants were required to be able to communicate in Arabic or English. Eligible participants included licensed pharmacists, clinical pharmacists, and pharmaceutical care leaders who were working or had previously worked in MOH hospitals in the Makkah Region. They were invited to participate irrespective of their knowledge of PMACs and the existence of PMACs at their hospitals. No minimum years of experience were required for participation, and eligible individuals were enrolled regardless of their employment status (full-time or part-time).

The study was limited to hospitals affiliated with the MOH, given their dominant presence and central role in providing public healthcare services in the Makkah Region. Additionally, MOH hospitals differ substantially from private and university hospitals in terms of budgeting, regulatory systems, and organizational readiness for service implementation [13]. This focus allowed for consistent comparison across study settings and ensured relevance to MOH-level policy and service planning. Individuals working in private or university hospitals, or outside the Makkah Region, were therefore excluded.

### 2.3. Participant recruitment

Two types of sampling strategies were employed: purposive and convenience sampling. Purposive sampling was used to select participants or data sources that addressed the research questions, involving directly inviting 50 pharmacists based on their professional roles and involvement in pharmaceutical care services. They were contacted through pharmaceutical work groups and via their work email addresses, obtained from the official hospital websites. Pharmaceutical care leaders were also asked to assist with recruiting and to suggest the names of pharmacists involved in PMAC services at their hospitals. In parallel, convenience sampling was used to recruit additional pharmacists and clinical pharmacists who had or did not have a clinic at their hospital [14]. Those participants were approached through social media, including Snapchat announcements, Facebook posts, and WhatsApp messages to MOH colleagues. Due to the open nature of social media dissemination, the total number of pharmacists reached through this method could not be quantified. Participants were informed that they could withdraw from the study at any time up to the conclusion of the interview. The recruitment and data collection period spanned from January 1, 2024, to February 28, 2024.

### 2.4. Study setting and location

The study was conducted in MOH hospitals in the Makkah Region, Saudi Arabia. The Makkah Region is a critical healthcare hub due to its large resident population. MOH hospitals were selected because they operate under a unified governance and funding system and represent the primary public healthcare provider in the region, making them an appropriate setting to explore system-level barriers and facilitators to PMAC implementation.

### 2.5. Data collection

#### Interviews.

Semi-structured interviews were conducted by phone in English. The interviews were carried out by an MOH employee (WLZ) with a master’s degree in clinical pharmacy. The interview guide was developed during meetings between the researchers after reviewing the literature and adapting the CFIR tool [1,10,11,15,16]. The draft guide was sent to three clinical pharmacists to validate its clarity, simplicity, and comprehensibility, and was modified before the data collection. The pharmacists were chosen for their substantial contributions to the field and the qualitative orientation of their studies. S1 File provides details of the interview guide.

#### Recruitment materials.

Before their interviews, the participants received an information sheet regarding the study and a consent form to sign. A form addressing their sociodemographic characteristics was completed at the start of the interview. To address concerns regarding anonymity, only background information, such as each participant’s age, sex, qualifications, and number of years of practice and PMAC existence, was collected.

#### Pilot study.

A pilot study using the developed questions and prompts was conducted with two participants. The following objectives guided the pilot study: (a) to validate the questions and assess whether they were sufficiently open-ended to generate relevant data in line with the study aims, (b) to ensure the interview guide was easy to understand, (c) to amend/reorganize the interview guide as necessary, and (d) to select the most suitable approach to transcription and test the transcription software.

#### Saturation.

Theoretical saturation was achieved at the sampling level, as the complete range of constructs identified in the literature was discussed with participants and reflected in the data [17]. At the data collection stage, data saturation was obtained, and new data tended to be redundant to those already collected. No new codes were generated after the eighth participant. Three more participants were interviewed to confirm the data saturation. Inductive and a priori thematic saturation were also achieved during the data analysis.

### 2.6. Data analysis

The interviews were audio recorded in English and transcribed using the intelligent verbatim method. Post-interview notes were taken to help analyze the data and amend the questions as appropriate. The participants’ data were anonymized using a unique code and number (identifiable by the interviewer only). All interview sets’ data were analyzed using a thematic analysis approach with NVivo software. Thematic analysis was conducted in several steps: (a) data familiarization, where the interviewer reviewed all recordings and identified initial codes aligned with broad themes from the interview guide, (b) development of sub-themes organized under the main themes, (c) identification of key analytic ideas by reviewing the generated codes, (d) organization of codes by related concepts to identify patterns across the data, and (e) refinement and review of themes to ensure they accurately represented the coded data and overall dataset.

Coding was performed inductively and deductively, in which the themes were strongly related to the data and emerged from the researchers’ preexisting knowledge and concepts [18]. Codes and themes were developed iteratively by two researchers through independent readings of the transcripts. All themes were reviewed and mapped onto the CFIR domains (inner setting, outer setting, pharmacists’ characteristics, and the implementation process) [11]. Discrepancies in coding were resolved through consensus discussion to ensure reliability. Interviews were conducted until data saturation was reached, with no new themes emerging.

### 2.7. Ethical consideration

This study adhered to the ethical principles outlined in the Declaration of Helsinki and complied with local institutional ethical guidelines. Ethics approval was obtained from the Umm Al-Qura University institutional review board in Makkah City (HAPO-02-K-012-2023-11-1893), prior to data collection. Written informed consent was obtained from all participants before their participation. The consent form was sent via email, and participants were required to read, sign (digitally or manually), and return it electronically prior to scheduling the interviews. To ensure the trustworthiness of the findings, several strategies were implemented: (a) a detailed description of the study context, methodology, and analytical frameworks was provided to enhance transferability and reproducibility, (b) a deliberate search for negative or deviant cases was considered in the analysis to enhance credibility, and (c) three types of triangulations were applied to enhance the trustworthiness, these are data triangulation, site triangulation, and investigator triangulation. Data triangulation was achieved by including diverse participant types (pharmacy leaders, clinical pharmacists, and regular pharmacists) recruited from multiple MOH hospitals in different geographical locations (site triangulation). This approach captured a range of perspectives and experiences, reducing the risk of bias associated with a single data source. Investigator triangulation was also employed by having two researchers independently code and analyze the interview transcripts, which helped minimize individual bias [19–21].

## 3. Results

A total of 12 pharmacists responded to the recruitment effort and met the inclusion criteria. Eleven pharmacists were interviewed; one pharmacist was unable to participate due to time constraints. The average length of the interviews was 20 ± 10 minutes, and the interviewer followed the same guide for all the interviews. The participants were pharmacists aged 31–40 years, representing both male and female pharmacists with varying professional roles and career stages. They worked in different MOH hospitals in the Makkah Region: Jeddah (n = 6), Makkah (n = 4), and Taif (n = 1). Seven participants were clinical pharmacists, two were pharmaceutical care leaders, and two were regular pharmacists. Four participants had implemented a PMAC in their working hospitals. S2 File outlines the participants’ relevant characteristics.

The thematic analysis revealed two core thematic categories: barriers and facilitators to implementing PMACs. The subtheme barriers were professional and interprofessional barriers, organizational and operational barriers, and patient-related barriers. In contrast, the facilitators’ sub-themes were workforce capacity and training, leadership and interprofessional support, organizational and patient support mechanisms, and infrastructure and digital resources.

### 3.1. Barriers to clinic implementation

#### 1. *Professional and interprofessional barriers.*

Participants described several professional and interprofessional barriers that influence the implementation of PMAC. A central challenge was the lack of professional recognition and trust from physicians, particularly specialists such as cardiologists and hematologists. One pharmacist described ongoing negotiations regarding pharmacists’ suitability to lead the clinic, noting persistent resistance from some medical specialties:

*“Before starting the clinic, there were some negotiations about whether we are the right people to manage the clinic, and the consultant did not support us …. now we have the same problem with the hematologists because we are just running the anticoagulants clinic for cardiology, but there is no anticoagulants clinic for internal medicine”, 03-PMAC clinical pharmacist*

The physicians’ resistance was also commonly reported during the early implementation phases, but diminished once they observed pharmacists’ competencies:

*“The first barrier was the physicians. There was a little resistance from the physician’s side because they did not understand until we explained to them thoroughly the importance of our role and our qualifications. They attended with us the first few clinics so that they were assured of what we were able to manage”, 01-PMAC clinical pharmacist*

In addition, participants highlighted insufficient organizational and leadership support, particularly during the shift to the virtual clinic.

*“There’s no motivation at all … the organization did nothing to support us, especially when we transferred to the virtual clinic. We did the whole work by ourselves, no one helped us except the health transformation staff; they supported the idea at the beginning, and that’s it”, 03-PMAC clinical pharmacist*

Another frequently reported barrier was the lack of pharmacists’ competencies (training and qualification) to run the clinic, limiting the service sustainability:

*“We still don’t have any consultants or cardio pharmacists … They are not qualified, it all depends on the hospital staff”, 01-Non-PMAC regular pharmacist*

#### 2. *Organizational and operational barriers.*

The healthcare system’s limitations in implementing PMACs included resource shortages, laboratory issues, and other issues related to the referral process.

*Resource shortages.* The shortage of trained clinical pharmacists and nursing support affected the clinic’s workflow and appointment organization:

*“If the pharmacist who runs the clinic goes on vacation, he has to find someone to cover his work”, 02-Non-PMAC clinical pharmacist*

*“We need the nurses to manage the clinic with us … to organize the patient appointments, to help the pharmacists in the clinic”, 03-PMAC clinical pharmacist*

Participants also reported limited and non-sustained training opportunities, with few refresher courses available for new staff. Moreover, inadequate clinic infrastructure, including the absence of a designated clinic area, frequent changes in clinic scheduling, and limited access to a computer, was perceived as negatively affecting the workflow and patient experience. Additional illustrative quotations supporting these findings are provided in the S3 File.

*Laboratory issues.* One clinical pharmacist described her experience of the lab system and its effect on the PMAC’s delivery.

*“The laboratory system was shut down two weeks ago, affecting the clinic. We couldn’t manage the clinic at that time because there were no results at all.” 03-PMAC clinical pharmacist*

Other pharmacists questioned the INR test’s sensitivity, the appropriateness of the time to measure the INR, and the accuracy of the results in some hospitals.

*“Patients usually have a problem doing INR levels at 8 am, with the clinic at 1 pm. They must wait in the hospital from morning to afternoon, which is a long time. Sometimes, we just say your INR level is okay, take your medication, and go home, especially if the patients are well-educated about their medications; it’s time-wasting.... We finish the clinic at 4 pm. Sometimes there are no results till we finish, and the patients will have waited since the morning.” 03-PMAC clinical pharmacist*

*Referral process.* Some participants highlighted issues with the referral process when transferring patients to different departments. For example, it was unclear who referred patients to the emergency department (ER) after they had developed complications or side effects.

*“The problem can only be the organization between us and the doctors. For example, if there is a side effect or bleeding and the patient needs admission to the ER, we should have an arrangement between us and the on-call doctors about the mechanism of patient admission.” 02-Non-PMAC clinical pharmacist*

The pharmacy staff also found the referral process to the PMAC unclear.

*“What is the mechanism for transferring a patient to the clinic? If the patient is discharged from the hospital, can he be referred to us automatically? Or will he first follow up with the cardio clinic and then be referred to us? So maybe this is the difficulty.” 02-Non-PMAC clinical pharmacist*

Some issues discussed related to arrangements with nursing staff, such as the coordination of PMAC and cardiology clinic appointment times and the type of clinic offered (i.e., in-person vs. virtual appointments).

*“Some of the issues stemming from the people managing the appointments in the hospital, are how the appointments are managed and how the message is sent to the patients. Some must visit the hospital, while others receive messages to attend the virtual clinic. There’s no clear way on how to use it and how to inform those patients.” 03-PMAC clinical pharmacist*

#### 3. *Patient-related barriers.*

Patients’ poor commitment and resistance to/rejection of the care provided in PMACs impacted the utilization and implementation of these clinics in MOH hospitals. This affected the workflow in PMACs, especially given the high number of patient referrals.

*“The resistance that comes from patients, not from physicians, is a barrier. The physicians knew how much the consultation clinic helped patients when we established it. But I am afraid of patients’ resistance. When they receive a clinic appointment, they might say: Why do I have to go? No need. I will go to the doctor.” 02-Pharmacy leader*

Such patient resistance was also reported for the virtual clinic.

*“When we transferred the clinic to the virtual clinic... some patients mentioned that they didn’t believe in the online or virtual clinic. They said we must come to see the doctor face-to-face to understand.” 03-PMAC clinical pharmacist*

### 3.2. Facilitators of PMAC implementation

#### 1. *Workforce capacity and training.*

Many respondents highlighted adequate clinical experience and targeted training as important enablers of PMAC implementation. They believed it should be comprehensive and of a professional standard to ensure a strong knowledge base. One suggested recommendation was to establish a dedicated training center for anticoagulant medications and to send staff from other sector hospitals to the training center to acquire advanced knowledge*:*

*“Send the staff to King Faisal Research Center Hospital for shadowing and training on how to run the clinic.” 01-PMAC clinical pharmacist*

#### 2. *Leadership and interprofessional support.*

Participants highlighted leadership engagement and interprofessional collaboration as key facilitators for the implementation of PMACs. The idea to establish PMACs was frequently described as originating from MOH leaders and cluster managers, whose endorsement, monitoring, and alignment with national priorities were perceived as critical enablers during implementation. Support was also provided at the hospital level by heads of clinical pharmacy services, pharmacy department leaders, hospital administrators, and other personnel facilitating the service delivery.

*“Currently, the MOH is working on an indicator of anticoagulation use in general. We are monitoring the use of anticoagulants via a survey. The MOH focused on this topic; we will have an anticoagulant clinic, which means there will be support from hospital administrators, pharmacy administrators, and the clusters.” 02-Pharmacy leader*

Pharmacists’ involvement in anticoagulation management helped reduce physicians’ workload and addressed medication-related issues beyond anticoagulation, including dose adjustment, administration, and adherence. Participants noted that PMACs were particularly beneficial for physicians from non-cardiology specialties with limited experience in anticoagulant management. Moreover, participants sought to establish good communication with other healthcare professionals through a shared anticoagulant committee, complemented by workshops and educational lectures.

*“If we could attend workshops with other healthcare professionals, clinical pharmacists, from other hospitals…. there are things that we discover individually, so, I prefer having a committee that brings together people in different specializations to share our new experience”, 03-PMAC clinical pharmacist*

#### 3. *Organizational and patient support mechanisms.*

The participants supported the need for clear MOH or hospital policies to implement and run the clinics.

*“To start any project, you must start from a good base, so the first thing you have to do is establish the policy and modify it per the hospital’s needs…. We don’t have a hospital policy right now, but we are working on it. We will use the MOH policy as a foundation and modify it based on our hospital setting.” 02-Pharmacy leader*

One clinical pharmacist explained their experience of developing a comprehensive hospital policy to run the PMAC:

*“We took the physician’s hospital policy regarding anticoagulants, and the pharmacy updated it after getting agreement from the cardiology physicians about adding the pharmacist managing part.” 01-PMAC clinical pharmacist*

Another clinical pharmacist indicated that they used international policies as a guide:

*“We tried to collect policies from different countries and used policies from the MOH and our experience... to create our own policy.” 03–PMAC clinical pharmacist*

#### 4. *Infrastructure and digital resources.*

The virtual clinic was also noted as one of the best solutions to overcome the barriers of space constraints, resource shortages, time constraints, and increased hospital visits. However, this type of clinic was considered most beneficial for well-educated patients who demonstrated good medication compliance.

*“We started the virtual clinic to overcome the problem of space or crowdedness among the patients outside the clinic.” 01–PMAC clinical pharmacist*

## 4. Discussion

This is the first in-depth national study on the barriers and facilitators to implementing PMAC services at MOH hospitals. The barriers identified in this study related to limitations in the healthcare system, pharmacists’ lack of support and expertise, and physician resistance. Patients’ poor commitment to follow-up or rejection of the care provided in the clinic were other barriers. In contrast, the main facilitators identified were the development of the healthcare system and support for professionals involved in PMAC provision. While CFIR and KTA were used to analyze the data and identify key barriers, they do not provide specific intervention strategies. Therefore, the Behavior Change Wheel (BCW) was used as an interpretive framework to conceptually map the identified barriers to potential behavioral domains. BCW is widely used in healthcare settings, including pharmacy practice, to develop behavior-change interventions [22,23]. Using the COM-B model, barriers were broadly categorized according to Capability, Opportunity, and Motivation. These categories interact in this “Behavior system” to produce the desired behavior, affecting these constituents [22]. This approach was intended to support interpretation of findings and generate hypotheses for future intervention development, rather than to prescribe specific implementation strategies. The intervention functions identified through BCW (e.g., training, environmental restructuring, persuasion, and incentivization) should therefore be considered exploratory and illustrative, particularly given the study’s qualitative and context-specific design [22].

In this study, barriers related to healthcare professionals included the lack of appreciation for the role of pharmacists by other healthcare providers. This barrier corresponds to the reflective motivation category in the BCW framework and contributes to a lack of trust between pharmacists and physicians. One study reported similar findings, in which pharmacists expressed negative feelings about properly utilizing their clinical skills in PMACs [24]. Current and previous findings suggest that pharmacists’ motivation to implement and continue running PMACs would increase if physicians in Saudi Arabia became more supportive of the clinics and their positive outcomes [25]. Given the emphasis on collaboration in the Saudi healthcare system, this support could be fostered through regular communication that clearly outlines the benefits of PMACs for improving patient care [26,27]. For example, engaging physicians in pharmacy staff meetings with presentations or distributing concise materials highlighting successful outcomes can help raise awareness of pharmacists’ roles and the advantages of the PMAC model [22,28].

Physicians’ resistance was another reported barrier categorized under psychological capability. The initial physician resistance may be rooted in broader systemic factors. For example, physicians in Saudi Arabia have primarily perceived pharmacists as dispensers of medications rather than having clinical roles [29]. Unclear role definitions and the limited integration of clinical pharmacy services within the MOH system may contribute to the undervaluation of pharmacists’ clinical competencies [29,30]. This perception has been reinforced by the limited availability of interprofessional education opportunities during medical and pharmacy training, resulting in inadequate exposure to collaborative practice models [31]. These systemic factors may explain why physicians initially lacked confidence in the PMAC model and why direct exposure to pharmacists’ clinical skills was necessary to overcome these preconceptions. However, our findings contradict those in the literature. For example, physicians in a previous New Zealand study had a good relationship with pharmacists. They suggested keeping the PMAC available for their patients, alongside strong support for a nationwide rollout [24]. Most participating physicians expressed confidence in the pharmacists’ management and the reliability of INR results. However, some did not refer medically unstable patients to the clinic. Similarly, most pharmacists believed that their relationships with physicians had strengthened after the implementation [24]. The variations in these results could be attributed to differences in culture and the appreciation and development of the pharmacist position (i.e., pharmacy as a career is still evolving in Saudi Arabia, despite a significant need for clinical pharmacists). To overcome physician resistance, our participants reported that pharmaceutical leaders had worked to increase physicians’ confidence in the PMAC by allowing them to attend the first few clinics to ensure the clinical pharmacists’ competencies in running the clinic. Previous Saudi studies have also suggested that increasing physicians’ awareness of the benefits of pharmacist involvement in medication management may help mitigate their resistance [28,29]. Regular workshops that define the roles of clinical pharmacists and showcase successful peer-positive outcomes of PMACs in contemporary healthcare settings can facilitate change. These activities align with BCW intervention functions: education, persuasion, and modeling [22,28,29]. Moreover, previous studies have suggested that providing new physicians with exposure or training related to health system models that integrate clinical pharmacy services, such as PMACs, may support their understanding and confidence in pharmacist-led care [28].

At the system level, this study identified several barriers, including clinical pharmacist shortages and a lack of training, which were mapped to the environmental opportunity component of the BCW framework. Similar challenges have been reported in a previous study, including a lack of independently trained and adequately qualified clinic staff [1]. In the literature, centralized, multidisciplinary approaches to anticoagulant management have been described as potential ways to address workforce limitations. Participants in the current study had a similar suggestion. In addition, both BCW theory and international studies support continuous education and training as relevant mechanisms for addressing skill gaps and a limited workforce capacity [1,22,25,32]. These findings should be interpreted as exploratory and intended to inform future research and service development rather than as definitive implementation recommendations.

In our study, most participants reported that the absence of a designated area for the clinic resulted in recurrent changes to the PMAC location, which impacted clinic workflows. Longer waiting times for patients requiring frequent follow-up/monitoring were also identified in one study as a barrier to current PMAC services and were categorized as an environmental opportunity [6,22]. The authors suggested locating a large PMAC in the heart of the cardiology clinic, accompanied by updated, computerized medical references and databases to provide the necessary support, resources, and access to patient information [6]. This solution is an enablement intervention function in BCW theory. Another solution suggested in the current study was the provision of a virtual clinic as an environmental restructuring intervention in BCW theory. Alanazi *et al.* supported this suggestion during the COVID-19 pandemic. They found that the median (IQR) TTR% for the in-person clinic was 54.6 (27.3), compared to 50 (33.3) for the virtual clinic, with no statistically significant difference in TTR between the two clinic types (P = 0.07). However, telemedicine was considered a positive strategy to reduce patients’ hospital visits and offered effective remote evaluation when patients were socially isolated during emergencies [33]. Another promising approach identified in a recent local study to address hospital overcrowding is the implementation of point-of-care testing (POCT) [34]. POCT enables rapid clinical decision-making in terms of diagnosis, treatment choice, and monitoring, offering efficacy, safety, and convenience [1,25,34,35]. This solution would require the environmental restructuring and modeling of the results [22]. The POCT may also overcome issues related to INR testing that were reported in this study and the literature, which are identified as environmental opportunity barriers. For example, Tadesse *et al.* found that inconsistent reporting of INR findings and patients’ extended waiting times were concerns that prevented patients from taking the test and forced them to obtain testing from different sources. Consequently, the results were difficult to interpret due to the different test calibration ranges and standards of practice among laboratories, which also impacted outcomes-based anticoagulant dosage recommendations [1].

Patient-related barriers were also highlighted in this study, particularly patients’ poor commitment to follow-up appointments and refusal of care. These were identified as psychological capability barriers to implementing PMAC, in contrast to findings reported in the literature. It was shown that PMACs fostered good relationships between pharmacists and patients, largely due to the sit-down nature of consultations and one-on-one time spent with patients. Patients have thus become more aware and appreciative of the role and capacity of pharmacists in managing their health [36]. Drawing on the BCW framework and existing literature, patient education and supportive environmental approaches have been described as potential mechanisms to address such challenges [22,37]. For example, reminder systems for follow-up appointments have been discussed in prior studies as illustrative strategies that may support continuity of care [37]. In the Saudi context, telehealth mobile applications (e.g., Seha, Mawid, Tawakklna) have been appreciated by patients as acceptable tools to facilitate the delivery of their care and remind them of their appointments [38]. However, public trust in such applications remains necessary. Building strong relationships with pharmacists and physicians (within the PMAC model) is key to fostering trust and increasing acceptance of telehealth applications and clinical pharmacy services [38]. These observations should be viewed as exploratory and intended to inform future research rather than to prescribe specific interventions. A summary of recommendations for practice and improvement strategies is provided in Table 1.

**Table 1:** Participants identified potential strategy domains mapped to barriers in PMAC implementation (exploratory analysis).

	Facilitators			
Barriers	Workforce capacity & training	Leadership & interprofessional support	Infrastructure & digital resources	Organizational & patient support mechanisms
**Professional and interprofessional barriers**				
Lack of pharmacist competencies	√			
Lack of pharmacist appreciation		√		
Lack of staff expertise	√	√	√	
Physician resistance	√	√		
**Organizational and operational barriers**				
Staff shortage and lack of training	√	√		
Clinic area and computers			√	
Lack of hospital policy		√		√
INR testing			√	
**Patient-related barriers**				
Patient resistance & poor commitment	√	√	√	√

Although this study primarily explored the barriers and facilitators from the pharmacists’ perspective, it is essential to acknowledge that other institutional factors may also impact the service implementation and success. For example, a previous local study highlighted how the healthcare setting and the high number of patients visiting the anticoagulation clinic could affect appointment availability, follow-up frequency, and consequently, patient outcomes [39]. Other Saudi studies have also reported that more intensive follow-up is required for patients at the initiation phase of warfarin therapy, those with uncontrolled INR, high thrombotic risk, or recent medication changes, all of which could affect treatment success if the PMAC has limited capacity [6,34,39]. Strong collaboration among healthcare providers has also been identified as a key contributor to the overall success of the PMACs [6]. Anticoagulation management requirements may also vary according to the type of anticoagulant used and influence PMAC implementation. For example, direct oral anticoagulants (DOACs) require different patient education and follow-up approaches compared with warfarin, which may lead to distinct barriers to their integration within PMACs. Barnes *et al.* (2019) reported that key barriers to integrating DOAC care in anticoagulation clinics include low provider awareness of DOAC-related services, financial challenges associated with providing care to DOAC patients, and ambiguity among clinical staff regarding the scope of DOAC care [16]. These findings suggest that incorporating DOAC-related workflows into PMACs may require tailored training and updated funding models to ensure effective service delivery.

This qualitative study identified key themes and perspectives on a broad topic that could not be obtained from the survey respondents. The research team considered triangulation and different techniques to ensure the trustworthiness of the findings. However, some limitations remained. First, despite employing both purposive and convenience sampling strategies and using multiple recruitment channels, including formal channels (e.g., professional work groups) and informal channels (social media platforms), this study may be subject to selection and nonresponse bias. Pharmacists who chose to participate were likely engaged and positive about the topic, which may have led to nonresponse bias and left some negative perspectives unexplored. Some pharmacists may have overlooked social media invitations, which could have further contributed to the limited diversity of perspectives. Second, the shared professional background between the interviewer and participants may have introduced a social desirability bias, as participants might have provided answers that they perceived as more professionally acceptable. Third, although data saturation was achieved and the qualitative approach provided valuable exploratory insights, this study was limited to pharmacists working in MOH hospitals in a single region (Makkah Region). Excluding private, military, and university hospitals and focusing on a small, pharmacist-only sample may limit the generalizability of the findings to other healthcare sectors/regions in Saudi Arabia, and warrants cautious interpretation of the results. In addition, representation across cities within the Makkah Region was uneven, which may have limited the capture of city-specific organizational or contextual differences. Moreover, perspectives on the implementation of PMAC may vary by pharmacists’ age, position, and career stage, which should be considered when interpreting the findings and assessing their transferability. Future studies should also include perspectives from other stakeholders, such as physicians, patients, and policymakers, to provide a more comprehensive understanding of PMAC implementation. Fourth, despite the use of two sampling strategies to enhance participation, the limited sample size may reflect cultural factors in Saudi Arabia, where qualitative interviews are not commonly used, and some pharmacists may be hesitant to participate or have their conversations recorded. Lastly, this study did not differentiate between patients using oral anticoagulants and those using DOACs. Future studies could explore whether barriers and facilitators differ according to the type of anticoagulant therapy, as patient experiences and management requirements may vary between different users.

## 5. Conclusion

This study explored the factors influencing the implementation of PMAC service from the perspectives of different stakeholders. The overarching barriers were related to limitations within the healthcare system, while patient-related barriers, such as poor acceptance and commitment to follow-up, were new findings. The literature supports the participants’ suggested solutions and aligns with BCW theory. While the findings are context-specific, they may inform similar initiatives in other MOH hospitals across Saudi Arabia, considering the uniformity of the healthcare system. Additionally, these insights help complete the KTA model’s action cycle by offering potential strategies for implementing, optimizing, and evaluating PMAC services. Further research is needed to explore the views of other stakeholders and quantify the impact of PMACs on both clinical and non-clinical patient outcomes. This could enhance the clinics’ recognition among healthcare professionals, accelerate their expansion, and support long-term sustainability.

## Supporting information

S1 File Participant’s Interview Guide. (PDF)

S2 File Participants’ characteristics (n = 11). (PDF)

S3 File Supplementary tables and quotations. (PDF)
