# Supplementary material for: Pharmacists’ perspectives on implementing pharmacist-managed anticoagulant clinics in Makkah Region Ministry of Health Hospitals: A qualitative study
Source: PLoS One. 2026 Feb 2;21(2):e0342079. doi: 10.1371/journal.pone.0342079 (PMC12863539; doi:10.1371/journal.pone.0342079)
Supplement: S1 File — (PDF) [file pone.0342079.s001.pdf]

Pharmacists' Perspectives on Implementing Pharmacist-Managed Anticoagulant Clinics in Makkah Region Ministry of Health Hospitals: A Qualitative Study

Supplementary file 1. Participant's Interview Guide.

1- Opening of the interview

- Greet the participants and thank them for taking part in the research.
- Explain again the purpose of the study.
- Ask participants if they would like to ask any questions before starting the interview.
- Emphasize to the participants that there is no right or wrong answer and that I am just interested in their experiences.
- Discuss the participants' information sheet if they have not read it beforehand.
- Discuss the participant's informed consent and ensure it is signed.
- Check the audio recorder and ask the participants if they are happy to begin the interview.

Before we start this interview, I would like to confirm that:

- Your participation in this study is completely voluntary.
- You are free to refuse to answer any question.
- You can withdraw, but only up to the interview's conclusion.
- The interview will be strictly confidential and anonymized, and all information disclosed during the interview will be only available to the research team.
- Excerpts from this interview may be part of the final report of the project. However, the information in the project report will NOT be linked to you.
- All reports and information collected will be stored securely at Umm Al-Qura University.

Are you ready to proceed with the interview?

2- Body of the interview and research questions

The research will be "*Exploring Barriers and Facilitators of Implementing Pharmacists-led Anticoagulants Clinics at Ministry of Health Hospitals in Makkah Region: A Qualitative Study*". The participants will be introduced to what I mean by "barriers and facilitators of implementing anticoagulant pharmacy clinic".

The interview will consist of four parts, questions for each group of participants (pharmacy leaders, clinical pharmacists, pharmacists), which are:

a. Knowledge of the anticoagulant pharmacy clinic.
b. Implementation process of anticoagulant pharmacy clinic in Makkah region.
c. Barriers to implementing anticoagulant pharmacy clinic in Makkah region.
d. Facilitators to implementing anticoagulant pharmacy clinic in Makkah region.

Interview Part	If an anticoagulant clinic is implemented	If an anticoagulant clinic is NOT implemented
**Knowledge of the anticoagulant pharmacy clinic**	1. How long have you known about the anticoagulant pharmacy clinic?<br><br>2. How well do you know the pharmacist's role in managing anticoagulant medication?<br>**Prompt:**<br>- What service could the pharmacists provide to the patients in the clinics?<br><br>3. How do you perceive the potential impact of the clinic on patient outcomes?	1. Have you heard of the anticoagulant pharmacy clinic?<br><br>2. **If the answer is (Yes),** How well do you know about the pharmacist's role in managing anticoagulant medication?<br>**Prompt:**<br>- What services could the pharmacists provide to the patients in the clinics?<br><br>3. **If the answer is (No),** In your opinion, what could be the role of pharmacists in managing patients taking anticoagulants medication?<br>**Prompt:**<br>- How do you think pharmacists can provide help to patients taking anticoagulants?<br><br>4. How do you perceive the potential impact of the clinic on patient outcomes?<br>**Prompt:**<br>- Or they do not need it because they receive enough information form their physicians?<br><br>5. Since studies support the positive outcomes of implementing the clinic, how long do you expect to establish the clinic in your hospital?<br>**Prompt:**<br>- Are you aware of any MOH plans to Implement the clinic in the hospitals of Makkah region?

**Implementation process**	**Since the clinic is established in your hospital:**	**Since you are working at an MOH hospital, and the clinic is not implemented:**
	1. When was the anticoagulant pharmacy clinic service applied?<br><br>2. What was the necessity to establish the clinic in your hospital?<br>**Prompt:**<br>- Did patients need to have the clinic? (e.g., to improve INR, reduce side effects)<br>- Does the clinic impact on the patient's outcomes? What are the outcomes? (e.g., quality of life, Hospital re-admission rate, Medication adherence)<br>- Considering the physician practices and their number at your hospitals, did they need to establish such a clinic?<br><br>3. What is your experience of implementing the clinic?<br>**Prompt:**<br>- What was the idea of applying the clinic?<br>- Who came up with the idea (from pharmacy staff, leader, or physician)?<br>- How was the clinic planned to open? (step or process of opening it).<br><br>4. How do you think the MOH policy would affect the clinic's implementation process?<br><br>5. How many specialized or trained pharmacists do/did you have at your hospital, who can manage the clinic?<br><br>6. What are the resources that were relied upon during the implementation of the clinic?<br>**Prompt:**<br>- Have you ever had a copy of policy specifications, training, or workshops?	1. Do you need such a clinic at your hospital?<br><br>2. What is the necessity of establishing the clinic in your hospital?<br>**Prompt:**<br>- Do patients need to have the clinic? (e.g., to improve INR, reduce side effects)<br>- Does the clinic impact on the patient's outcomes? What are the outcomes? (e.g., quality of life, Hospital re-admission rate, Medication adherence)<br>- Considering the physician practices and their number at your hospitals, do they need to establish such a clinic?<br><br>3. How do you think the MOH policy would affect the clinic' implementation process?<br><br>4. Do you have a specialized or trained pharmacist at your hospital who can manage the clinic? How many?<br><br>5. What are the resources that could you rely upon to establish the clinic?<br>**Prompt:**<br>- Do you need a copy of the policy specification, and training on how to run the clinic?

**Barriers of anticoagulant pharmacy clinic**	1. What are the barriers you faced while implementing the clinic or the **barriers/obstacles** that can stop running the clinic?<br>**Prompt:**<br>- Have you ever faced any issues with providing the service? (technical problems, workload, laboratory issues and physician resistance).<br><br>- What is your opinion of the pharmacist's knowledge and training to counsel the patients and run the clinic?<br><br>- What do you think about the availability of the resources to implement the clinic? Are they sufficient?<br>e.g., staff shortage, time, rooms (space), computer, money (financial support).	1. In your perspective what are the **barriers** you will face while implementing the clinic?<br>**Prompt:**<br>- Any issues with providing the service? (technical problems, workload, laboratory issues and physician resistance).<br><br>- What is your opinion of the pharmacist's knowledge and training to counsel the patients and run the clinic?<br><br>- What do you think about the availability of the resources to implement the clinic? Are they sufficient?<br>e.g., staff shortage, time, rooms (space), computer, money (financial support).
**Facilitators of anticoagulant pharmacy clinic**	1. What were the **facilitators** that supported the opening or the continuity of the clinic?<br>**Prompt:**<br>What are the types of support you received? e.g.,<br>- motivation,<br>- organizational support (by head of hospital/MOH),<br>- supportive leadership,<br>- employing skilled staff,<br>- providing training, workshops,<br>- perceived effectiveness of the clinic (like good follow up results, patient satisfaction).	1. What do you think the **facilitators** that would support the opening or the continuity of the clinic?<br>**Prompt:**<br>What types of support would you need to implement the clinic in your hospital?<br>- motivation,<br>- organizational support (by head of hospital/MOH),<br>- supportive leadership,<br>- employing skilled staff,<br>- providing training, workshops,<br>- perceived effectiveness of the clinic (like good follow up results, patient satisfaction)

3- Closing the interview:

- Ask the participants if they would like to add or ask about anything else before closing the audio recorder and finishing the interview.
- Thank the participant again for taking part in the study.
