# Supplementary material for: Pharmacists’ perspectives on implementing pharmacist-managed anticoagulant clinics in Makkah Region Ministry of Health Hospitals: A qualitative study
Source: PLoS One. 2026 Feb 2;21(2):e0342079. doi: 10.1371/journal.pone.0342079 (PMC12863539; doi:10.1371/journal.pone.0342079)
Supplement: S2 File — (PDF) [file pone.0342079.s002.pdf]

Pharmacists' Perspectives on Implementing Pharmacist-Managed Anticoagulant Clinics in Makkah Region Ministry of Health Hospitals: A Qualitative Study

Supplementary file 2. Participants' characteristics (n = 11).

Variable		N (%)
Sex	Male<br>Female	3 (27.2)<br>8 (72.7)
Age	31 – 40	11 (100)
Hospital location	Jeddah<br>Makkah<br>Taif	6 (54.5)<br>4 (36.4)<br>1 (9.1)
Availability of the PMAC at the hospital	Yes<br>No	4 (36.4)<br>7 (63.6)
Current position	Pharmacist<br>Clinical pharmacist<br>Pharmacy leader	2 (18.1)<br>7 (63.6)<br>2 (18.1)
Working experience	< 10 years<br>> 10 years	3 (27.2)<br>8 (72.7)
Experience in Anticoagulant therapy	Yes<br>No	4 (36.4)<br>7 (63.6)
Employment status	Full time<br>Part time	10 (90.9)<br>1 (9)
Department/service	Inpatient pharmacy<br>Cardiology ward<br>Anticoagulant clinic<br>Head of Pharmacy Department<br>Head of Pharmaceutical Care services	1 (9.1)<br>3 (27.2)<br>4 (36.4)<br>1 (9.1)<br>2 (18.2)
Highest qualification	Bachelor's degree<br>Master's degree<br>Residency program<br>PhD	1 (9)<br>7 (63.6)<br>2 (18.1)<br>1 (9)
