# Supplementary material for: Pharmacists’ perspectives on implementing pharmacist-managed anticoagulant clinics in Makkah Region Ministry of Health Hospitals: A qualitative study
Source: PLoS One. 2026 Feb 2;21(2):e0342079. doi: 10.1371/journal.pone.0342079 (PMC12863539; doi:10.1371/journal.pone.0342079)
Supplement: S3 File — (PDF) [file pone.0342079.s003.pdf]

Pharmacists' Perspectives on Implementing Pharmacist-Managed Anticoagulant Clinics in Makkah Region Ministry of Health Hospitals: A Qualitative Study

Supplementary file 3. Supplementary table and quotations.

Table S1. Barriers to implementing PMAC related to healthcare providers.

Barrier	Comment	Quotation
Lack of appreciation	The lack of appreciation from other healthcare providers, like cardiologists and hematologists, was a barrier experienced by pharmacists delivering care in the anticoagulant clinic.	*"Before starting the clinic, there were some negotiations about whether we are the right people to manage the clinic, and the consultant did not support us .... now we have the same problem with the hematologists because we are just running the anticoagulants clinic for cardiology but there is no anticoagulants clinic for internal medicine", 03-PMAC clinical pharmacist*
	It is believed that this lack of appreciation to the pharmacy staff resulted in a lack of trust between pharmacists and physicians.	*"There's no trust between the consultants and the clinical pharmacists to manage the clinic, this is one of the barriers", 03-PMAC clinical pharmacist*
Lack of support	Pharmacists delivering the care in the clinic did not receive enough support and motivation from the pharmacy or MOH leaders.	*"There's no motivation at all ... the organization did nothing to support us, especially when we transferred to the virtual clinic. We did the whole work by ourselves, no one helped us except the health transformation staff; they supported the idea at the beginning and that's it", 03-PMAC clinical pharmacist*
Lack of expertise	It was believed that the implementation process would take time due to having insufficient trained or qualified clinical pharmacists.	*"We still don't have any consultants or cardio pharmacists ... They are not qualified so, it all depends on the hospital staff", 01-Non-PMAC regular pharmacist*
Lack of understanding of the service benefits	This was noticed by one pharmacist who denied the need for the clinic.	*"In hospitals, there is a pharmacist covering each department. He reviews these things, so it does not require a clinic", 02-Non-PMAC regular pharmacist*
Physician resistance	Most respondents reported physician resistance to the idea of the clinic. However, it was clarified that this barrier exists at the beginning of the clinic implementation, after that the barrier diminished.	*"The first barrier was the physicians. There was a little resistance from the physician's side because they did not understand until we explained to them thoroughly the importance of our role and our qualifications. They attended with us the first few clinics so that they were assured of what we were able to manage", 01-PMAC clinical pharmacist*

Abbreviations: PMAC = pharmacist-managed anticoagulant clinic; MOH = Ministry of Health.

Table S2. Resource shortage barriers to implementing PMAC.

Barrier	Comments	Quotation
Staffing problem	Shortage of clinical pharmacists was one of the obstacles affecting the workflow. Such barrier was highlighted, especially when the pharmacy staff needed to have vacations or emergency leaves.	*"If the pharmacist who runs the clinic goes on vacation, he has to find someone to cover his work, or if any emergency condition happens and he cannot cover the clinic, he has to ask another pharmacist to cover", 02-Non-PMAC clinical pharmacist*
	A shortage of nursing staff who is assigned to organize the clinic workflow.	*"We had some issues with nurses' staff because in the actual clinic we need the nurses to manage the clinic with us ... to organize the patient, appointments, to help the pharmacists in the clinic", 03-PMAC clinical pharmacist*
Training	Additional training courses on running the clinic were needed. Courses were mainly provided to the pharmacy staff at the beginning of the implementation, but there were no follow-up or refresher workshops.	*"There is not much courses and educational materials, it was just one course provided by the ASHP and MOH. So, the new commers they don't have any certificates or training", 01-PMAC clinical pharmacist*
Clinic area and computers	Participants agreed that there was no designated area for the clinic. They perceived that the mismanagement of the clinic area affected the workflow through crowdedness due to an inappropriate area or scheduled time.<br><br>There were also issues related to the availability of sufficient computers to run the PMAC.	*"At the beginning, they (administrative staff) gave us a clinic and then they moved the clinic to another place ... so we lost the computers and the staff that we had in the last clinic .... they changed the date of the clinic from Sunday to Thursday which confused the patients, and patients were making complaints to the MOH" 03-PMAC clinical pharmacist*

Abbreviations: PMAC = pharmacist-managed anticoagulant clinic; ASHP = American Society of Health-System Pharmacists; MOH = Ministry of Health.

Table S3. Facilitators of running and implementing PMAC.

Facilitator	Comments	Quotation
Workforce capacity and training	Most participants emphasized the need for clinical pharmacists (staff expertise) to manage the clinic, especially in cardiology, due to their close contact and strong relationships with physicians.	*"Clinical pharmacist is the best one that the physician trusts because of their awareness and more understanding ... clinical pharmacist is the best person to take this responsibility", 02-Non-PMAC clinical pharmacist*
Physician's support	This support was observed to contribute to a decrease in physicians' workload and time spent in the clinic with their patients. Clinical pharmacists helped them explore inquiries beyond the anticoagulant side effects and complications like dose adjustment, method of administration, and adherence.	*"Physicians want the clinic to reduce the load ... We have many doctors from different departments like Internal medicine; these doctors are not good in anticoagulants, and most patients use the anticoagulant as prophylaxis or treatment doses. This increases the load on cardiology doctors if the patient comes off surgery and is given a prophylaxis dose. I give him enoxaparin, but he does not know how to use it", 01- Non-PMAC clinical pharmacist*
Communication between healthcare providers	Participants expressed a need for shared platforms, such as multidisciplinary anticoagulation committees, workshops, and joint educational activities, to enhance coordination, standardize practices, and promote shared learning across institutions.	*"If we could attend workshops with other healthcare professionals, clinical pharmacists, from other hospitals, in a workshop that brings us together…. there are things that we discover individually, so, I prefer having a committee that brings together people in different specializations to share our new experience", 03-PMAC clinical pharmacist*
